# Folic acid as a potential therapeutic agent for Alzheimer's disease: Effects on inflammatory cytokines, amyloid deposition, and neurotransmitter metabolism

**DOI:** 10.5937/jomb0-57713

**Published:** 2025-10-28

**Authors:** Shaowei Jing, Yanqiu Wang, Yang Liu, Yi Luo, Xiaoqing Wen, Yao Ma, Haoxuan Zhu, Gongcai Chen, Xiaochun Ouyang

**Affiliations:** 1 Department of Neurology, No 908th Hospital of Chinese PLA Joint Logistic Support Force, Nanchang, Jiangxi, 330000, China; 2 Department of Respiratory and Critical Care Medicine, No 908th Hospital of Chinese PLA Joint Logistic Support Force, Nanchang, Jiangxi, 330000, China; 3 Department of Gerontology, No 908th Hospital of Chinese PLA Joint Logistic Support Force, Nanchang, Jiangxi, 330000, China

**Keywords:** Alzheimer's disease, inflammatory cytokines, folic acid, neurotransmitters, Ab1-42, randomized controlled trial, Alchajmerova bolest, inflamatorni citokini, folna kiselina, neurotransmiteri, Ab1-42, randomizovana kontrolisana studija

## Abstract

**Background:**

Alzheimer's disease (AD) is a degenerative disease of the central nervous system characterized by neuroinflammation and amyloid deposition. Folic acid (FA), a B vitamin, may improve the course of AD by modulating inflammation and neuroprotection. This study aimed to investigate the effects of FA supplementation on serum inflammatory cytokines (IL-1b, IL-6, TNF-a), amyloid (Ab1-42), Tau proteins, and neurotransmitters (GABA, 5-HT, Ach) in AD patients.

**Methods:**

We conducted a follow-up-controlled trial; 114 AD patients were included and randomly divided into a control group (donepezil treatment) and an experimental group (donepezil + FA treatment) for 3 months. Inflammatory factors, Ab1-42, Tau, neurotransmitter levels and nutritional status were assessed before and after treatment.

**Results:**

The total effective rate of the experimental group (89.47%) was significantly higher than that of the control group (75.44%), and the levels of inflammatory factors (IL-1b, IL-6, and TNF-a), Ab1-42, and Tau were significantly lower (P&lt;0.05), and neurotransmitters (GABA, 5-HT, and Ach) and nutritional indexes (albumin and hemoglobin) were substantially higher.

**Conclusions:**

FA supplementation can effectively delay AD progression by inhibiting neuroinflammation, reducing amyloid deposition, regulating neurotransmitter metabolism and improving nutritional status.

## Abstract (sr)

**Uvod:**

Alchajmerova bolest (AD) je degenerativna bolest centralnog nervnog sistema koja je karakterisana neuroinflamacijom i nakupljanjem amiloida. Folna kiselina (FK), vitamin B grupe, može poboljšati tok AD modulišući inflamaciju i neuroprotekciju. Cilj ove studije bio je da se istraže efekti suplementacije FK na serumske inflamatorne citokine (IL-1b, IL-6, TNF-a), amiloid (Ab1-42), Tau proteine i neurotransmitere (GABA, 5-HT, Ach) kod pacijenata sa AD.

**Metode:**

Sproveli smo kontrolisanu studiju praćenja u kojoj je učestvovalo 114 pacijenata sa AD, koji su nasumično podeljeni u kontrolnu grupu (lečenje donepezilom) i eksperimentalnu grupu (lečenje donepezilom + FK) tokom 3 meseca. Inflamatorni faktori, Ab1-42, Tau, nivoi neurotransmitera i nutritivni status procenjivani su pre i posle lečenja.

**Rezultati:**

Ukupna efektivnost eksperimentalne grupe (89,47%) bila je značajno viša nego u kontrolnoj grupi (75,44%), dok su nivoi inflamatornih faktora (IL-1b, IL-6 i TNF-a), Ab1-42 i Tau bili značajno niži (P&lt;0,05), a nivoi neurotransmitera (GABA, 5-HT, i Ach) i nutritivni indeksi (albumin i hemoglobin) bili su značajno viši.

**Zaključak:**

Suplementacija FK može efikasno usporiti napredovanje AD inhibiranjem neuroinflamacije, smanjenjem nakupljanja amiloida, regulisanjem metabolizma neurotransmitera i poboljšanjem nutritivnog statusa.

## Introduction

Alzheimer’s disease (AD) is a degenerative disease of the central nervous system with a very high incidence in the elderly population [1]. Statistics show that the global incidence of AD is approximately 5.2 per cent in people over 65 years of age and more than 30 per cent in people over 85 years of age [2]. At present, the pathogenesis of AD is not fully understood, and the deterioration of body function and nutrient loss caused by increasing age are considered to be the main risk factors [3]. Therefore, in the treatment of AD, it is necessary to pay attention not only to the pathological changes of patients but also to the improvement of their nutritional status [4].

Folic acid (FA) is a water-soluble vitamin from the vitamin B family, with its parent compound consisting of pteridine, para-aminobenzoic acid, and glutamic acid [5]. In clinical practice, FA supplementation has been shown to have the effect of nourishing nerve cells and reducing cerebrovascular damage due to harmful substances. It is often used as a nutritional supplement for pregnant women during pregnancy to protect the neurological function of newborns [6]. Studies have shown that FA is efficiently transported across the blood-brain barrier (BBB), especially in the choroid plexus, via specific transporters (e.g., RFC, PCFT) or receptor-mediated endocytosis (e.g., folate receptor alpha, FRα) [7]. Therefore, in cerebrovascular diseases, the use of FA is also effective in reducing blood lipids and blood viscosity and preventing the occurrence of cerebrovascular obstruction [8]. These findings suggest the potential value of our FA application in AD. Markun et al. [9] also demonstrated that FA supplementation can better improve human neurological function and relieve depressive symptoms and fatigue in humans.

The pathogenesis of Alzheimer’s disease is closely associated with neuroinflammation and synaptic dysfunction. Key inflammatory cytokines such as IL-1β, IL-6, and TNF-α are elevated in AD brains, driving microglial activation and neuronal damage [10]. Aβ1-42 aggregates trigger neurotoxic cascades, whereas hyperphosphorylated Tau activates kinase activity, leading to microtubule disassembly, impaired axonal transport, and consequent cognitive decline [11] [12] [13]. Additionally, neurotransmitter imbalances – particularly deficits in GABA (inhibitory signalling), 5-HT (mood regulation), and Ach (memory formation) – are hallmarks of AD progression [14]. These biomarkers collectively reflect disease severity and therapeutic potential. FA, through its role in one-carbon metabolism and antioxidant pathways, may modulate these pathological processes by reducing homocysteine (Hcy)-mediated toxicity and enhancing Aβ clearance [15] [16], positioning it as a promising adjunct therapy for AD.

However, we found that current studies on FA mainly focus on the effect on the clinical efficacy of patients, and the observation of the impact on patient’s immune inflammatory factors is rarely seen. Given the above limitations, this study will analyze the influence of FA on the immunoinflammatory factors, Aβ1-42, and Tau of AD patients to provide more comprehensive reference and guidance for the future clinical treatment of AD.

## Materials and methods

### Research participants

According to the sample size estimation results of G-Power software (set effect size to 0.3, alpha to 0.05, and power to 0.95), it was learned that we needed a minimum of 111 study subjects. 114 AD patients admitted to No 908th hospital of the Chinese PLA Joint Logistic Support Force from March 2023 to August 2024 were selected as the research subjects. They were randomized into a control group and an experimental group of 57, each using a random number table method and received conventional treatment and conventional treatment plus supplemental FA, respectively. All patients were unaware of their subgroups. The hospital Ethics Committee approved the study protocol without reserves (No. XXX), and all of the study subjects’ immediate families signed informed consent forms. In addition, the research was conducted strictly following the Declaration of Helsinki.

### Inclusion and exclusion criteria

Inclusion criteria: Confirmed diagnosis of AD by comprehensive examinations such as haematology, electroencephalography, and neuropsychology; Age 65 years old; Complete medical records. Exclusion criteria: Drug allergies; Serious heart, brain, kidney, and other organ diseases; Malignant tumours; Thyroid diseases and severe malnutrition (weight more than 20% below normal); Inability to actively cooperate with the research to complete the treatment and follow-up.

### Methods

The control group was given oral donepezil hydrochloride (Eisai China Inc., H20050978) orally, with an initial dose of 5 mg/time once a day; the dose was increased to 10 mg/time one month later. Based on the above treatment, the experimental group was treated with oral FA Tablets (Beijing Silian Pharmaceutical Co., Ltd., H10970079), 5 mg/time, once a day. Both groups were treated for 3 months. Otherwise, the clinical interventions were consistent in both groups.

### Clinical efficacy evaluation

Clinical efficacy was assessed after treatment using the Mini-Mental State Examination (MMSE) [17]. The MMSE consists of a series of questions covering a wide range of tests in orientation, memory, attention and numeracy, recall, and language skills. Markedly effective is defined as a significant improvement in symptoms such as memory loss and communication impairment and a >75% reduction in the MMSE score. Effective corresponds to a significant improvement in symptoms, with a reduction rate of 75% and 45% in the MMSE score. Ineffective is defined as a slight improvement in symptoms, with a <45% reduction in the MMSE score [18].

### Clinical testing

Fasting venous blood was collected before and after treatment from both groups of patients, and the serum was obtained by centrifugation. Albumin (ALB), haemoglobin (HGB), and total protein (TP) were detected by an automatic biochemical analyzer (MAGLUMI X3, VEDENG), and -aminobutyric acid (GABA), 5-hydroxytryptamine (5-HT), and acetylcholine (Ach) were measured by radioimmunoassay. Interleukin-1β (IL-1β), Interleukin-6 (IL-6), Tumor necrosis factor-α (TNF-α), Aβ1-42, and Tau were detected using enzyme-linked immunosorbent assay (ELISA) assays, and the kits were purchased from Beijing Quanshijin Biotechnology Co.

### Endpoints

(1) Clinical efficacy was assessed and compared. (1) Immunoinflammatory factors, Aβ1-42, and Tau. (3) Neurotransmitters (GABA, 5-HT, and Ach) and nutritional proteins (ALB, HGB, and TP) before and after treatment were analyzed.

### Statistical analysis

This study used SPSS25.0 for statistical analysis. The data were checked for normal distribution (Shapiro-Wilk test). The chi-square test was used to compare the count data [n(%)]. To identify the statistical significance of measurement data (x̄±s), the independent sample t-test and paired t-test were used. P<0.05 was the significance threshold.

## Results

### Comparison of clinical data

As shown in Table 1, the inter-group comparison of age, sex, and other data showed no statistical significance (P>0.05), indicating that the subjects in the two groups were comparable.

**Table 1:** There was no difference in clinical data between the two groups.

Groups<br>(n=57)	Gender		Age	Body mass<br>index (BMI)<br>(kg/m^2^)	Duration of<br>disease<br>(years)	MMSE score	Smoking	
	male	female					yes	no
Control	34 (59.65%)	23 (40.35%)	71.46±5.20	21.30±1.89	3.39±1.40	12.12±2.66	22 (38.60%)	35 (61.40%)
Experimental	30 (52.63%)	27 (47.37%)	70.98±6.13	21.00±2.16	3.42±0.96	12.04±3.27	20 (35.09%)	37 (64.91%)
χ^2^ (or t)	0.570		0.445	0.788	0.156	0.157	0.151	
P	0.450		0.657	0.433	0.876	0.876	0.698	

### Comparison of clinical efficacy

The statistical results of clinical efficacy after treatment, shown in Table 2, revealed that the treatment effective rate was 89.47% in the experimental group and 75.44% in the control group. The intergroup comparison showed a higher total effective rate in the experimental group compared to the control group (P=0.049), suggesting higher treatment efficacy in the experimental group.

**Table 2:** Experimental group had better clinical efficacy.

Groups (n=57)	Markedly effective	Effective	Ineffective	Total effective rate (Markedly<br>effective+effective)
Control	20 (35.09%)	23 (40.35%)	14 (24.56%)	75.44%
Experimental	26 (45.61%)	25 (43.86%)	6 (10.53%)	89.47%
χ^2^				3.881
P				0.049

### Comparison of serum inflammatory cytokines, Aβ1-42 and Tau

After treatment, serum inflammatory cytokines, Aβ1-42 and Tau were reduced in both groups, in which IL-1β, IL-6, TNF-α, Aβ1-42 and Tau were lower in the experimental group than in the control group (P<0.05), that is to say, the experimental group had less inflammation after treatment (Table 3).

**Table 3:** Experimental group had a milder inflammatory response. Note: vs before treatment, *P<0.05.

Groups<br>(n=57)	IL-1β (ng/L)		IL-6 (ng/L)		TNF-α (mg/mL)		Aβ1-42 (ng/L)		Tau (ng/L)	
	Before	After	Before	After	Before	After	Before	After	Before	After
Control	32.21±<br>4.13	30.54±<br>3.30*	140.53±<br>15.16	120.03±<br>13.35*	6.72±<br>0.68	5.69±<br>0.96*	224.40±<br>11.46	200.40±<br>17.06*	230.74±<br>21.53	206.95±<br>26.86*
Experimental	33.63±<br>4.71	26.76±<br>3.16*	138.55±<br>17.70	110.43±<br>22.18*	6.63±<br>0.79	4.72±<br>0.59*	226.10±<br>16.68	191.86±<br>16.14*	224.40±<br>25.12	186.22±<br>28.59*
t	1.720	6.231	0.644	2.801	0.686	6.507	0.632	2.743	1.447	3.988
P	0.088	<0.001	0.521	0.006	0.494	<0.001	0.589	0.007	0.151	<0.001

### Comparison of neurotransmitters

As shown in Table 4, the two groups were not statistically different in the neurotransmitter detection results before treatment (P>0.05); GABA, 5-HT, and Ach in both groups increased after treatment, with more significant increases in the experimental group (P<0.001), indicating better neurological function in the experimental group after treatment.

**Table 4:** Experimental group had better neurological function. Note: vs before treatment, *P<0.05.

Groups<br>(n=57)	GABA (μmol/L)		5-HT (ng/mL)		Ach (nmol/L)	
	Before	After	Before	After	Before	After
Control	19.65±3.30	24.18±2.54*	44.72±5.41	58.35±6.94*	23.09±2.78	32.01±6.00*
Experimental	18.87±2.03	30.25±3.73*	43.35±7.52	69.37±5.25*	22.88±3.06	40.80±8.05*
t	1.508	10.150	1.113	9.556	0.379	<0.001
P	0.134	<0.001	0.268	<0.001	0.706	<0.001

### Comparison of nutritional status

As shown in Table 5, the two groups were similar in pre-treatment levels of nutritional proteins (P>0.05). After treatment, the nutritional proteins in the control group remained unchanged (P>0.05), while the ALB, HGB, and TP in the experimental group increased (P<0.05).

**Table 5:** Experimental group had a better nutritional status. Note: vs before treatment, *P<0.05.

Groups<br>(n=57)	ALB (g/L)		HGB (g/L)		TP (g/L)	
	Before	After	Before	After	Before	After
Control	40.25±6.65	39.58±7.43	112.21±16.13	113.43±15.26	70.78±10.46	70.78±9.68
Experimental	41.31±6.02	45.65±5.48*	116.14±15.76	121.48±16.58*	69.62±13.32	75.34±8.14*
t	0.888	4.968	1.318	2.698	0.520	2.721
P	0.376	<0.001	0.190	0.008	0.605	0.008

## Discussion

As a primary degenerative encephalopathy, AD has an insidious onset and is often difficult to detect at the initial stage [19]. As the disease progresses, the neurological and cognitive functions of patients are generally irreversibly damaged, resulting in an adverse prognosis, limitations in daily life, and even endangering patients’ life safety in severe cases [20]. In this article, the treatment effect of the experimental group was significantly improved compared to the control group, fully demonstrating the positive impact of FA supplementation on AD patients.

In addition, we observed that IL-1β, IL-6, TNF-α, Aβ1-42 and Tau were lower in the experimental group than in the control group after treatment, confirming that FA has an excellent anti-inflammatory effect. The reason may be that FA activates PPAR to increase the transcription of IDE, which increases the degradation of Aβ1-42, so its blood concentration is reduced accordingly [21]. Aβ includes two main subtypes, Aβ1-40 and Aβ1-42, of which Aβ1-42 has the strongest neurotoxicity, is prone to aggregation and formation of amyloid, and promotes the formation of senile plaques, which is the most closely related to AD [22]. Tau belongs to microtubule-associated proteins, which is a phosphorylated protein in the normal brain, maintaining the stability of the microtubule assembly process and connecting the axonal microtubules [23]. Still, it is abnormally over-phosphorylated in the brains of patients with AD, losing the normal function of vascular transport and forming plaques that are deposited in the cerebral vasculature, leading to behavioural and cognitive dysfunction in the patients [24]. In this study, the significant decrease in Tau levels in the experimental group also suggests that our FA may affect the phosphorylation level of Tau, thus alleviating the pathological manifestations of AD patients.

As we all know, Hcy is an essential pathological factor in AD, with its elevated levels directly related to pathological progression in AD patients [25]. Huang X et al. [26] mentioned that FA deficiency inhibits methylenetetrahydrofolate reductase (MTHFR) activity and reduces 5-MTHF production, which leads to impaired Hcy remethylation and Hcy accumulation. FA supplementation has been shown to effectively promote plasma conversion of Hcy to reduce plasma Hcy concentrations in patients with metabolic syndrome [27], which is speculated to be one of the pathways for FA to alleviate AD. In addition, FA can effectively inhibit the apoptosis of cerebral ischemic nerve cells and enhance the activity of free radical scavenging enzymes in vivo, thereby inhibiting free radical production and lipid peroxidation and ultimately reducing cranial nerve damage [28]. This can also be confirmed by the comparison of neurotransmitter levels between the two groups of patients, where the experimental group had higher levels of GABA, 5-HT, and Ach than the control group.Normal neurotransmitters can ensure information transmission between neurons and maintain brain physiological functions [29]. Therefore, FA supplementation can contribute to neurotransmitter metabolism and improve neurological function, and its combination with donepezil can further improve the effect of AD treatment.

It is well known that the synthesis of neurotransmitters depends on specific amino acids [30]. The main source of amino acids is through nutritional intake and metabolism of nutrient proteins [31]. At the same time, some antioxidant nutrients (e.g., vitamin E, selenium, zinc) have been shown to reduce free radical damage to neurons and protect the transmitter system [32]. In the subsequent comparison of nutritional status, the experimental group showed an increase in ALB, HGB, and TP, as well as a reduced risk of malnutrition, demonstrating that FA is also of great significance in improving the nutritional status of AD patients. Malnutrition has been shown to be one of the risk factors for AD [33]. A cross-sectional survey by Gómez-Gómez et al. [34] also showed significantly worse nutritional status in AD patients with cognitive impairment. It can be seen that there is a mutual influence and regulation between nutritional status and the pathological progression of AD. The main effects of FA on nutritional status are as follows: (1) FA can promote the maturation of bone marrow blasts. FA deficiency, on the other hand, may lead to an increase in immature cells in the body, resulting in macrocytic anaemia and leukopenia [35]. (2) The synthesis of FA and DNA can promote brain development and maintain brain function [36]. (3) FA has the function of nourishing cranial nerves [37]. (4) FA can increase appetite, promote digestion, enhance spleen and stomach functions, promote nutrient absorption, and reduce the risk of nutrient loss [38]. (5) FA helps the human body absorb sugars and amino acids and metabolize excess proteins, and it is also essential for cell growth [39]. Based on the above effects, FA supplementation is effective in enhancing the nutritional status of AD patients.

However, due to the small number of cases in this study and the short follow-up time, it is notexcluded that the analysis results may be accidental. Besides, more outcome measures should be considered further to evaluate the overall impact of FA on AD and provide a more reliable reference for clinical practice. In addition, due to the lack of support from in vitro assays, we were unable to determine the specific effect of FA on protein metabolism for the time being. To address this situation, we will initiate a study to verify this as soon as possible.

## Conclusion

FA supplementation can effectively improve the neurological function and inflammation response of AD patients, inhibit the expression of Aβ1-42 and Tau, and improve their nutritional status. These findings suggest that FA is a potential therapeutic drug for AD with some clinical applications and helps to improve the health of AD patients.

## Dodatak

### Availability of data and materials

Original data in this study are available from the corresponding author upon reasonable request.

### Acknowledgements

Not applicable.

### Authors’ contribution

Xiaochun Ouyang and Gongcai Chen designed the study, Shaowei Jing and Yanqiu Wang wrote and revised the manuscript, Yang Liu and Yi Luo collected and analyzed data, Xiaoqing Wen and Yao Ma visualization of data, Haoxuan Zhu supervised the study, Shaowei Jing and Yanqiu Wang made equal contributions in this work as co-first authors. All authors read and approved the final submitted manuscript.

### Conflict of interest statement

All the authors declare that they have no conflict of interest in this work.
